# Mechanisms of Weight Regain following Weight Loss

**DOI:** 10.1155/2013/210524

**Published:** 2013-04-16

**Authors:** Erik Scott Blomain, Dara Anne Dirhan, Michael Anthony Valentino, Gilbert Won Kim, Scott Arthur Waldman

**Affiliations:** ^1^Division of Clinical Pharmacology, Department of Pharmacology and Experimental Therapeutics, Thomas Jefferson University, Philadelphia, PA 19107, USA; ^2^Department of Nutrition, College of Health Science, West Chester University, West Chester, PA 19383, USA

## Abstract

Obesity is a world-wide pandemic and its incidence is on the rise along with associated comorbidities. Currently, there are few effective therapies to combat obesity. The use of lifestyle modification therapy, namely, improvements in diet and exercise, is preferable over bariatric surgery or pharmacotherapy due to surgical risks and issues with drug efficacy and safety. Although they are initially successful in producing weight loss, such lifestyle intervention strategies are generally unsuccessful in achieving long-term weight maintenance, with the vast majority of obese patients regaining their lost weight during followup. Recently, various compensatory mechanisms have been elucidated by which the body may oppose new weight loss, and this compensation may result in weight regain back to the obese baseline. The present review summarizes the available evidence on these compensatory mechanisms, with a focus on weight loss-induced changes in energy expenditure, neuroendocrine pathways, nutrient metabolism, and gut physiology. These findings have added a major focus to the field of antiobesity research. In addition to investigating pathways that induce weight loss, the present work also focuses on pathways that may instead prevent weight regain. Such strategies will be necessary for improving long-term weight loss maintenance and outcomes for patients who struggle with obesity.

## 1. Introduction

Obesity is a global public health crisis. Its incidence continues to rise, and the prevalence of overweight has outstripped that of underfed [1]. The prevalence of overweight and obesity has increased over the past three decades [2]. Wang et al. predict that if rates continue in this fashion, by the year 2030, 86.3% of adults will be overweight or obese and 51.1% will be obese [2]. According to the 2009-2010 National Health and Nutrition Examination Survey (NHANES), 78 million (35.7%) US adults and 12.5 million (16.9%) US children and adolescents were obese [3]. In addition to being a widespread health problem on its own, obesity is a risk factor for the development of chronic diseases such as coronary heart disease, type 2 diabetes, hypertension, dyslipidemias, stroke, and cancer [4–6]. In fact, the deleterious effects of obesity are greater than both smoking and drinking in terms of overall health conditions and health-related costs [7]. In 2008, costs to treat obesity totaled $147 billion in the US [8]. 

The etiology of obesity is believed to be multifactorial, with both genetic and environmental contributions. A key determinant of obesity is the balance between ingested calories and the body's basal energy expenditure. Obesity therefore results when small positive energy balances accumulate over a long period of time [9, 10]. Presently, of the one-third of Americans who are obese, an estimated 50%–70% are trying to lose weight [11–13]. Lifestyle modification, which generally consists of a combination of nutrition, physical activity, and behavioral modification is an oft-used strategy to help patients achieve weight loss and maintenance [14, 15]. In their study to identify strategies associated with losing 5% and 10% of body weight among obese US adults, Nicklas et al. reported that the most popular strategies among obese participants trying to lose weight were eating less, exercising more, eating less fat, and switching to lower-calorie foods [16]. However, conventional weight loss strategies such as diet and exercise as well as medical therapy have not shown effectiveness in treating severe obesity in the long term [17]. The only known effective treatment for severe obesity is bariatric surgery, also known as weight loss surgery. Bariatric surgery has been shown to dramatically improve or resolve comorbidities associated with obesity, such as type 2 diabetes, hypertension, sleep apnea, and dyslipidemia [18]. 

Despite the efficacy of bariatric surgery, lifestyle modification therapies may be preferable to surgery since many patients are poor candidates for the operation and some may suffer postsurgical complications [19]. This difficulty, coupled with the limited efficacy of targeted obesity therapeutics, makes lifestyle modification a potentially favorable intervention [17]. However, a major barrier exists to its efficacy: weight regain. This regain is a common occurrence in patients who have lost weight by implementing lifestyle modifications, regardless of the dietary or behavioral intervention used. Approximately 30%–35% of lost weight is regained one year following treatment and 50% of patients will return to their baseline weight by the fifth year out from weight loss [20]. According to NHANES (1999–2006), a mere 1 in 6 overweight and obese adults reported maintaining weight loss of at least 10% for 1 year, at any point in their lives [21]. 

Body weight, feeding, and energy metabolism are regulated by a variety of hormones derived from the gut, adipose, and other tissues. These distinct signals are centrally integrated by the hypothalamus, which responds through key neuroendocrine axes that regulate energy expenditure, activity level, and feeding [22]. It has been speculated that the body cannot distinguish between dieting and starvation and, as such, the body's first response to weight loss is to prevent adverse effects of starvation [22]. The body exerts profound changes on the neuroendocrine milieu, promoting weight regain [22]. The present review discusses recent research highlighting these weight loss-induced alterations promoting weight regain in obese patients.

## 2. Energy Expenditure and Nutrient Metabolism

Energy expenditure varies according to changes in body weight. Consequently, weight fluctuation may be a key player in weight maintenance once weight is lost. Resting energy expenditure (REE) is defined as the energy needed to fuel minimal daily functions of cells and organs [23, 24]. Total energy expenditure (TEE) represents a compilation of expenditures, including resting metabolic rate, diet-induced thermogenesis, and activity-related energy expenditure [25]. Higher body weights are associated with a much higher TEE and a higher total energy intake [9]. Weight loss leads to a decrease in REE [26], which accounts for approximately 60 percent of TEE in humans [27]. Following weight loss, a decrease in REE is thought to lead to weight gain [28], suggesting a vicious cycle of obesity, followed by weight loss, followed by weight regain, followed by obesity, and so on. The concept of weight loss leading to a decrease in energy expenditure is known as “adaptive thermogenesis” and evidence suggests that these changes increase hunger, thus promoting weight regain [27]. Therefore, a difficulty in treating obesity may stem from the metabolic and physiologic responses resisting the maintenance of the decreased body weight.

The Prevention of Obesity Using Novel Dietary Strategies (POUNDS LOST) study funded by the National Heart, Lung, and Blood Institute compared the effects of four different diets (two low-fat, high carbohydrate diets and two high-fat, low carbohydrate diets) on weight loss and further compared the weight loss to changes in energy expenditure in 811 overweight or obese individuals over 24 months. REE measurements were taken at baseline, 6 months, and 24 months. At baseline, REE was higher for men than for women, and there was a strong positive correlation between body weight and REE. At 6 months, REE decreased significantly with weight loss, and this has been proposed to be a contributing factor in weight regain. REE increased back to baseline at 24 months, when weight regain was still less than 50% in these patients. These findings suggest that a good portion of weight regain occurs within the two years while REE is decreased, but that regain continues even after REE returns to normal. This is consistent with epidemiologic evidence that demonstrates weight regain beyond 24 months [20]. It is therefore unclear what role this return to baseline REE at 24 months plays in weight regain. 

There is also significant evidence that the composition of the diet used for weight loss influences REE and weight regain. Major macromolecule nutrients such as carbohydrate, protein, and fat in food stimulate oxygen consumption differently [26]. This in turn may influence changes in body weight and possibly weight regain [26]. Several studies have shown that high-protein, low-carbohydrate diets are effective for short-term weight loss, but evidence remains sparse in studying successful long-term weight loss and weight maintenance [29–31] The POUNDS LOST study examined the effect of diet composition on body weight and REE but found that both were unaffected by diet composition [26]. Ebbeling et al. randomly assigned obese patients to one of the three diets over four weeks: low fat, low glycemic index, and very low carbohydrate [28]. Among the participants who lost 10% to 15% baseline weight, REE decreased the least in the very low carbohydrate group, suggesting that a low-carbohydrate diet may deter weight regain [28]. Furthermore, another study compared five diets that differed in protein content and glycemic index in order to determine the best diet to prevent weight regain over a 26-week period [32]. Results from this study found that the high-protein, low-glycemic index diet was best for maintenance of weight loss [32]. Consistent with these findings, one group studying diet-induced obese rats observed a shift from fat to carbohydrate metabolism that occurred during weight regain [33]. Furthermore, another diet-induced rat obesity study found that if rats are maintained on a low carbohydrate, high fat diet and subsequently switched to a standard chow diet, they gained much more weight than the rats which were maintained on the standard chow diet throughout the study [34]. Future studies should be carried out over a longer duration in order to further evaluate a role for low-carbohydrate diets in the prevention of weight regain. Energy restriction and weight loss is believed to involve a shift in metabolism that favors lipid oxidation over carbohydrate, with later weight regain coinciding with a metabolic reversal back to carbohydrates [33, 35]. Thus, a low carbohydrate diet may protect against weight regain by blocking this metabolic reversal.

## 3. Neuroendocrine Adaptations Opposing Weight Loss

Although initially promising, the discovery of the hormone leptin and its contribution to obesity in the leptin knockout mouse (*ob/ob*) did not prove to be a “quick fix” to the problem of obesity. Despite observations in wild type and *ob/ob* mice that leptin supplementation decreases feeding and weight gain, there is little evidence to support leptin deficiency as an etiologic factor in sporadic human obesity [36–39]. Indeed, leptin levels in humans are correlated with weight gain and therefore *increase* in obesity, and evidence suggests that resistance to leptin's anorexigenic effects plays a role in this phenomenon [40–44]. In addition to leptin, a multitude of other neuroendocrine signals influence food intake and body weight. This ever-expanding list of appetite-mediating hormones includes leptin, ghrelin, cholecystokinin (CCK), peptide YY (PYY), insulin, pancreatic polypeptide (PP), glucagon-like peptide 1 (GLP-1), and uroguanylin [45–52]. These hormonal signals are integrated by the hypothalamus, which anchors various neuroendocrine axes including the hypothalamic-pituitary-thyroid axis and the hypothalamic-pituitary-adrenal axis. These axes in turn contribute to body weight homeostasis [53, 54]. Given the central role that these pathways play in the regulation of appetite, energy expenditure, and body weight, it is not surprising that this neuroendocrine milieu is profoundly altered in response to obesity and subsequent weight loss.

Recently, an important study by Sumithran et al. investigated the compensatory regulation of satiety hormones following weight loss in obese patients [55]. The investigators enrolled 50 overweight or obese patients and subjected them to a significant weight loss regimen over 10 weeks. Levels of circulating satiety hormones were measured at week 10, and then again at week 62 to assess long-term persistence of hormonal changes. Importantly, weight loss in obese patients resulted in significant and persistent reductions in levels of leptin, peptide YY, cholecystokinin, and insulin and increases in levels of ghrelin and pancreatic polypeptide. All of these hormonal changes are believed to be permissive of weight regain (with the exception of increased pancreatic polypeptide) and these observations are consistent with the increased subjective appetite reported by subjects. Indeed, the investigators report a strong linear correlation between the observed decreases in leptin and weight regain in patients. In another study, Varady et al. also showed a significant decrease in leptin levels following weight loss of over 5%, but not below 5%, suggesting that a critical level of weight loss is necessary to induce compensatory changes [56]. These findings are in accordance with other recent literature that demonstrated blunted release of the satiety hormones PYY and GLP-1 secondary to weight loss [57, 58]. 

In addition to altering levels of key satiety hormones, weight loss also has been shown to be associated with changes in several key hypothalamic-pituitary neuroendocrine axes that may promote weight regain. The thyroid hormone axis has long been associated with energy expenditure, and weight gain is a common clinical presentation in patients with hypothyroidism [59]. The hypothalamus secretes thyrotropin releasing hormone (TRH) which induces secretion of thyroid stimulating hormone (TSH) into the circulation by the anterior pituitary [60]. This in turn causes the thyroid gland to secrete thyroid hormones T3 and T4, which are involved in regulation of body temperature and energy expenditure, possibly by regulation of uncoupler proteins in the mitochondrial apparatus [61]. Consistent with this role as a mediator of energy expenditure, TSH has been shown to be inversely proportional to resting energy expenditure (REE), and feedback from T3 and T4 drives down TSH and increases energy expenditure [62–64]. Importantly, weight loss and dietary energy restriction has been shown to suppress the thyroid hormone axis in many studies [65–69]. Although the durability of this response during the natural history of weight regain remains unclear, this adaptive response would reduce REE and be permissive of weight regain. 

The hypothalamus also regulates body weight through the hypothalamus-pituitary-adrenal axis, which regulates cortisol levels in the circulation. Clinically, cortisol excess in humans (exemplified by Cushing syndrome) is associated with obesity [70]. This proobesity effect is at least partially due to suppression of leptin-induced satiety [71]. Importantly, cortisol levels increase markedly with energy restriction in a number of human studies [72–75]. This represents yet another mechanism by which the hypothalamus responds to weight loss and activates pathways that promote weight regain. These findings advance our understanding of weight regain by highlighting the importance of alterations to the neuroendocrine milieu in the compensatory response following weight loss.

## 4. Gastrointestinal Motility

The roles that gastric emptying and gastrointestinal motility play in obesity and weight loss are largely unclear and the evidence is sometimes contradictory. Some evidence suggests that obese individuals may exhibit accelerated gastric emptying, although the opposite has been shown as well [76–80]. Increased emptying may decrease the negative feedback induced by mechanical stretching and the presence of nutrients in the stomach. This would allow the consumption of larger and more frequent meals [81]. Consistent with this hypothesis, patients with pathologic delayed gastric emptying (as seen in gastroparesis and dyspepsia) exhibit dramatically decreased feeding and early satiety [82, 83]. However, a competing hypothesis suggests that less severe delayed gastric emptying in obesity may actually promote feeding through delayed delivery of nutrients to the duodenum and the associated release of satiety hormones. Recently, new evidence has emerged that this latter mechanism may in fact contribute to weight regain in obese patients [84]. 

Several studies have shown that weight loss induces a delay in gastric emptying, although others report no change [77, 84–87]. Interestingly, among the studies that documented a difference, Verdich et al. examined the time course of gastric emptying following weight loss. Although overall gastric emptying was unaffected, the investigators reported a delay in early emptying in the first 30 minutes after a meal [84]. The authors suggest that this early delay in the delivery of nutrients to the duodenum may delay the release of satiety hormones from the duodenum and promote feeding. Additional studies in gastric bypass patients provide further evidence for the permissive role of gastric emptying in weight regain. Increased transit time through the stomach is one of the hallmarks of gastric bypass, and as such it makes a good model of increased gastric emptying. Importantly, postprandial levels of the gut-derived satiety hormones PYY and GLP-1 are blunted in patients who lost weight through energy restriction but are increased in gastric bypass patients [57, 58, 88, 89]. Therefore, delayed gastric emptying seen in energy-restricted weight loss prolongs feeding by suppressing satiety hormones, while gastric bypass surgery accelerates gastric emptying and increases secretion of such hormones. These findings suggest a permissive role of gastric emptying in weight regain, although more studies into the exact nature of this relationship are necessary.

## 5. Subjective Appetite

Appetite represents the integration of biochemical, mechanical, neurological, and psychological pathways that regulate food intake into a single output: the desire to eat. Therefore, an increase in subjective appetite accompanies the described physiologic changes following weight loss that promote weight regain [35]. Indeed, multiple studies have documented this phenomenon, and these increases in appetite are sustained [55, 70]. Evidence suggests that additional changes occur to appetite beyond a simple increase in the desire for food. Cameron et al. showed that the perceived reward properties of food increase following weight loss, encouraging greater consumption [90]. Another study compared taste preferences between lean and formerly obese patients by having them rate different nutrient solutions based on taste [91]. Notably, lean subjects preferred a solution low in fat and sugar, while the formerly obese group preferred a solution high in both fat and sugar. Such a solution was extremely calorie dense and obesogenic. Therefore, weight loss induced overeating and preference for higher calorie foods. These changes would promote weight regain and represents the summation of all the compensatory mechanisms discussed previously.

## 6. Conclusion

The evidence presented in this paper provides novel insights into the nature of weight loss and associated compensatory changes. Further, it suggests that multiple redundant mechanisms have evolved to maintain body weight and thereby counteract a reduction in caloric intake. This redundancy may in part explain the high degree of failure and weight regain observed following weight loss through lifestyle intervention [20]. In this context, it is noteworthy that low-dose leptin replacement monotherapy has been shown to reverse many of the neurologic, hormonal, and behavioral adaptations that promote weight regain, despite the heterogeneous etiologies underlying weight regain [92–94]. These findings, coupled with the ongoing clinical development of targeted therapies to many other satiety pathways, lay the foundation for future strategies that combine lifestyle modification to induce weight loss with pharmacological intervention to prevent weight regain [95].

## Abbreviations

REE: Resting energy expenditure
NHANES: National Health and Nutrition Examination
*ob/ob* mice: Leptin knockout mice
TEE: Total energy expenditure
POUNDS LOST study: The Prevention of Obesity Using Novel Dietary Strategies study
CCK: Cholecystokinin
PYY: Peptide YY
PP: Pancreatic polypeptide
GLP-1: Glucagon-like peptide 1
TRH: Thyrotropin releasing hormone
TSH: Thyroid stimulating hormone.
